# EXAMINING PHYSICAL HEALTH AMONG COHORTS OF CENTENARIAN SURVIVORS IN THE HEALTH AND RETIREMENT STUDY

**DOI:** 10.1093/geroni/igac059.1205

**Published:** 2022-12-20

**Authors:** Rotem Arieli

**Affiliations:** Iowa State University, Bellevue, Washington, United States

## Abstract

This research highlights cohort differences in physical health among centenarians/near-centenarians in the Health and Retirement Study. Across 14 waves, participants aged 98 or older (n=494) were compared by three cohorts (i.e., 1890-1900, 1901-1910, and 1911-1920). Cohorts were examined at respective waves where participants were 96, 98, and 100 years of age. One-way ANOVA results presented significant differences in functional health at age 96 (F[2,324]=5.01, p<.01), subjective health at age 98 (F[2,212]=7.94, p<.001), and health conditions at ages 98 (F[2,213]=12.52, p<.001) and 100 (F[2,115]=3.45, p<.05). The oldest cohort had significantly better functional health than the youngest cohort (age 96), better subjective health than the two younger cohorts (age 98), and fewer health conditions than the youngest cohort (ages 98 and 100). Consistently, the oldest cohort performed better on subjective and objective health markers, providing implications for health care, disease prevention, and policy related to the shrinking “healthspan” among exceptional agers.

